# Risk Factors and Neuropsychological Assessments of Subjective Cognitive Decline (*plus*) in Chinese Memory Clinic

**DOI:** 10.3389/fnins.2019.00846

**Published:** 2019-08-14

**Authors:** Lixiao Hao, Yue Xing, Xuanyu Li, Bin Mu, Weina Zhao, Gubing Wang, Ting Wang, Jianguo Jia, Ying Han

**Affiliations:** ^1^Department of Geriatrics, Xuanwu Hospital of Capital Medical University, Beijing, China; ^2^Department of General Practice, Xuanwu Hospital of Capital Medical University, Beijing, China; ^3^Radiological Sciences, Division of Clinical Neuroscience, Queen’s Medical Centre, The University of Nottingham, Nottingham, United Kingdom; ^4^Department of Neurology, Xuanwu Hospital of Capital Medical University, Beijing, China; ^5^Department of Neurology, Hongqi Hospital of Mudanjiang Medical University, Mudanjiang, China; ^6^Faculty of Industrial Design Engineering, Delft University of Technology, Delft, Netherlands; ^7^Department of General Practice, School of General Practice and Continuing Education of Capital Medical University, Beijing, China; ^8^Department of General Surgery, Xuanwu Hospital of Capital Medical University, Beijing, China; ^9^Beijing Institute of Geriatrics, Beijing, China; ^10^National Clinical Research Center for Geriatric Disorders, Beijing, China; ^11^Center of Alzheimer’s Disease, Beijing Institute for Brain Disorders, Beijing, China

**Keywords:** Alzheimer Disease, subjective cognitive decline, risk factors, neuropsychological assessment, objective cognitive features

## Abstract

**Background:**

Since subjective cognitive decline (SCD) was standardized in 2014, many studies have investigated its features. However, the risk of SCD (*plus*) progressing to AD is much higher, and yet there have been few studies reporting the risk factors and neuropsychological assessment characteristics of SCD (*plus*).

**Objective:**

To characterize SCD (*plus*) by comparing it with normal control (NC), amnesic mild cognitive impairment (aMCI), and Alzheimer Disease (AD) regarding their demographics, lifestyle, family history of dementia, multimorbidity and the neuropsychological assessments.

**Methods:**

A total of 135 participants were recruited, including 23 NC, 30 SCD (*plus*), 45 aMCI and 37 AD. Descriptive statistics were provided. A logistic regression model was used to analyze the affecting factors of SCD (*plu*s), and finally the Receiver Operating Characteristic (ROC) analysis was applied to distinguish between SCD (*plus*) and NC.

**Results:**

(1) SCD (*plus*) group was younger than both the aMCI group and AD group. It consisted of more participants with mental work and higher body mass index (BMI) than the AD group. (2) Scores of Auditory Verbal Learning Test - Immediate recall (AVLT-IR) and AVLT-Long delayed recall (AVLT-LR) decreased in the following order: NC→SCD (*plus*)→aMCI→AD. (3) The Area Under Curve (AUC) for discriminating SCD (*plus*) and NC group was from 0.673 to 0.838.

**Conclusion:**

Aging is an important risk factor of both NC progressing to SCD (*plus*), and SCD (*plus*) progressing to aMCI or AD. In addition to aging, lower education level and lower BMI were significantly associated with greater odds of SCD (*plus*) progressing to aMCI or AD patients, whereas mental work was a protective factor of SCD (*plus*) progressing to AD. Finally, AVLT is a sensitive indicator of the cognitive decline and impairment in SCD (*plus*) in relative to normal controls.

## Introduction

World Alzheimer Reports showed that there were 47 million people living with dementia worldwide in 2016 (Alzheimer’s Disease International, 2016). This number is expected to increase to more than 131 million by 2050. The total estimated worldwide cost for dementia is US$ 818 billion, and by 2018 dementia will become a trillion-dollar disease. However, no effective modifying therapy has been validated yet, even for mild cognitive impairment (MCI) (Sperling et al., 2011).

Pre-mild cognitive impairment subjective cognitive decline (pre-MCI SCD), which has been defined as a self-experienced persistent decline in cognitive capacity in comparison with a previously normal status and objective cognitive performance within normal ranges (Jessen et al., 2014), is the first symptomatic manifestation of Alzheimer Disease (AD) and has received increasing attention (Rabin et al., 2015; Fernandez-Blazquez et al., 2016; Vogel et al., 2016). Individuals present with several specific features (e.g., age at onset of SCD ≥60 years; complaints about SCD within the past 5 years; the complainers feel their performance are not as good as their peers and concerning associated with SCD, etc.) associated with pre-MCI SCD would be identified as SCD (*plus*) (Jessen et al., 2014)^*^ – one of the preclinical stages of AD [See the appendix for the details of clinical features of SCD (*plus*)]. To our best knowledge, the risk of progressing to AD is higher for SCD (*plus*) than for SCD (Jessen et al., 2014). Accurate identification of SCD (*plus*) is therefore fundamental and crucial for early and successful intervention, which may help slow down its progression to AD and improve the prognosis.

Previous studies on SCD demographics showed people with SCD were younger and had a higher education than patients with MCI and AD dementia (Jonker et al., 2000; Garcia-Ptacek et al., 2014). Women were reported to be more likely to have SCD than men (Jonker et al., 2000). Less physical activity, hypertension, smoking and depression were found to be associated with the increase occurrence of SCD (Paradise et al., 2011; Chen et al., 2014). Also, among workers, those with cognitively demanding work were more sensitive to the changes of cognitive decline, and were thus more likely to report SCD (Rijs et al., 2015). Moreover, Aarts et al. (2011) demonstrated that SCD was related with multiple comorbidities (e.g., diabetes mellitus, stroke/transient ischemic attack, myocardial infarction, etc.). Cerebral trauma, middle-aged obesity, marital status (unmarried and widowed), born in the countryside, low social contact, and daily drinking were regarded as the risk factors for cognitive decline (Ao and Liu, 2004; Williams et al., 2010; Deng, 2014; Hao et al., 2017). In addition, a few studies showed that lower body mass index (BMI) associated with sarcopenia, was closely linked with the development of AD (Sugimoto et al., 2016; Ogawa et al., 2018). However, all these studies focused either on SCD or pre-MCI SCD, and little is known about the risk factors for SCD (*plus*) as a different category of patients.

Subjective cognitive decline may not a demonstrate detectable objective impairment when using the standardized neuropsychological assessment (Rabin et al., 2017), but it is still unknown about whether those assessments are useful for identifying SCD (*plus*). In addition, many studies have shown the distinction between SCD and MCI or AD, by using these neuropsychological assessments, such as MoCA, CDT and AVLT (Fernaeus et al., 2014; Zhao et al., 2015; Vyhnalek et al., 2017; Wang et al., 2019). Their high sensitivity and specificity have also been reported previously, suggesting that those assessments were able to distinguish SCD from MCI and AD (Vyhnalek et al., 2014; Huang et al., 2018; Park et al., 2018; Xu et al., 2018). However, little is known about the diagnostic power of these tools in discriminating SCD (*plus*) and NC. Furthermore, according to the diagnosis framework of SCD or SCD (*plus*), like NC, their scores of objective neuropsychological assessments are within the normal range. However, the chance of SCD (*plus*) progressing to MCI or dementia was significantly higher than the normal controls. Therefore, the distinguishing features between the SCD (*plus*) and NC groups are of great significance to study, as these could be more practically important for identifying people with SCD (*plus*) at an early stage and facilitate early intervention.

Therefore, our current study aims to (1) examine the relationship between SCD (*plus*) and the following potential risk factors including: demographics, family history of dementia, comorbidities, history of cigarettes smoking and drinking, and (2) to assess the sensitivity of the standard neuropsychological assessments on detecting SCD (*plus*) by comparing its scores with normal controls (NC), amnesic mild cognitive impairment (aMCI) and AD dementia. The ultimate purpose is to characterize SCD (*plus*) in order to provide more information for its early identification and intervention.

## Materials and Methods

### Ethics Statement

This study was approved by the Medical Ethics Committee of Xuanwu Hospital of Capital Medical University, Beijing, China. Written informed consent was obtained from either participants or their legally agreed surrogates.

### Participants

One Hundred thirty-five right-handed, Han Chinese subjects, including 30 SCD (*plus*), 45 aMCI, and 37 AD patients were recruited from the memory clinic of the Neurology Department, Xuanwu Hospital, Capital Medical University, Beijing, China from December 1, 2010 to June 30, 2016. 23 NC subjects were recruited from the hospital by advertisements.

### Assessment and Diagnosis Procedure

All of the subjects underwent a series of standardized clinical evaluations, including the Chinese version of Mini-Mental State Examination (MMSE) (Katzman et al., 1988), Montreal Cognitive Assessment (MoCA)- the Beijing version (Lu et al., 2011), Clinical Dementia Rating Scale (CDR) (Morris, 1993), World Health Organization-University of California Los Angeles Auditory Verbal Learning Test (AVLT) (Maj et al., 1994), Hachinski Ischemic Index (HIS) (Hachinski et al., 1975), Hamilton Anxiety Scale (HAMA) (Tang, 1984), and Hamilton Depression Scale (HAMD) (Hamilton, 1980).

Inclusion criteria for each group: All SCD (*plus*) subjects met the criteria for SCD (*plus*) proposed by SCD-I (Jessen et al., 2014): (a) presence of self-perceived continuous memory decline within the last 5 years, confirmed by informant report; (b) onset age ≥ 60 years old; (c) feeling cognitive decline worse than their peers and concerned about SCD; (d) normal performance on both MMSE, MoCA and AVLT after age and education adjustment; (e) CDR score = 0; (f) no impairment of daily life activities; and (g) HIS score ≤4.

The aMCI patients met the following criteria (Petersen, 2004): (a) memory complaint, preferably confirmed by an informant; (b) objective memory impairment; (c) normal or near-normal performance on general cognitive function with no or minimum impairment of daily life activities; (d) CDR score = 0.5; (e) HIS score ≤4; and (f) failure to meet the criteria of dementia according to DSM-IV (American Psychiatric Association, 1994).

The diagnosis of AD fulfilled standardized diagnostic criteria (McKhann et al., 1984; American Psychiatric Association, 1994; Dubois et al., 2007): (a) met the diagnostic criteria of dementia; (b) gradual and progressive decline in memory function over more than 6 months; (c) impaired episodic memory on objective testing; (d) HIS score ≤4; and (e) hippocampal atrophy confirmed by structural MRI.

Criteria of NC was defined as: (a) having no report of any cognition complaint; (b) normal performance on MMSE, MoCA and AVLT after age and education-adjusted; (c) CDR score = 0; and (d) no impairment of daily life activities.

Exclusion criteria for all the subjects: (a) a history of stroke (HIS >4); (b) severe depression and anxiety (HAMD >30, and HAMA ≥29); (c) other CNS diseases which could cause cognitive decline (e.g., brain tumors, Parkinson’s disease, encephalitis, or epilepsy); (d) other systemic diseases which could cause cognitive decline (e.g., thyroid dysfunction, severe anemia, syphilis, or HIV); (e) a history of psychosis or congenital mental growth retardation; (f) cognitive decline caused by traumatic brain injury; or (g) those who could not complete neuropsychological tests or with contraindication to MRI.

### Statistical Analysis

We conducted all analyses using the Statistical Package for the Social Sciences version 17.0 (SPSS Inc., Chicago, IL, United States). Descriptive statistics (sociodemographic characteristics, lifestyle, comorbidities, family history of dementia and scores of neuropsychological assessments) were calculated by percentages or median. The χ2 or Kruskal–Wallis test was used to assess group differences between SCD (*plus*) and the other three groups (NC, aMCI and AD group). For the four groups comparison, *p* < 0.05 was considered to be statistically significant, and corrected *p*′ value (*p* < 0.007) was used in the Partitions of Pearson’s chi-square statistics for *post hoc* pairwise comparisons. To examine the potential risk factors for each group in relation to the SCD (*plus*) group, we performed multiple logistic regression analysis with the removed backwards approach by including the sociodemographic characteristics, lifestyle, comorbidities and family history of dementia as the independent variables, and diagnosis as the dependent variable. In addition, odds ratios (ORs) were calculated for each variable. *p* < 0.05 was required for variables to be remained in the model. Finally, we obtained the receiver operating characteristic (ROC) curves and calculated the area under curve (AUC) of the characteristic factors that distinguish the NC and SCD (*plus*) group.

## Results

### Clinical Characteristics of the Participants

The clinical characteristics of the total sample were summarized in Table 1.

**TABLE 1:** Clinical characteristics of the study sample.

**Characteristics**	***N* = 135 *n* (%)**
Age, years	
≤75	96(71.1)
>75	39(28.9)
Gender	
Males	55(40.7)
Females	80(59.3)
Education, years	
≥12	94(69.6)
<12	41(30.4)
Job category	
Mental work	79(58.5)
Physical work	56(41.5)
BMI, Kg/m^2^	
≤23.9	83(61.5)
>23.9	52(38.5)
Lifestyle	
Current smoking	13(9.6)
Current drinking	18(13.3)
Family history of dementia	22(16.3)
Comorbidities	
Hypertension	48(35.6)
Cerebrovascular Disease	24(17.8)
Heart disease	12(8.9)
Diabetes	25(18.5)
Hyperlipidemia	40(29.6)
Multimorbidity	43(31.9)

*BMI, body mass index; Multimorbidity, accompanied with two or more diseases above.*

### Comparisons Between NC, SCD (*plus*), aMCI, and AD Groups

The difference between age among the four groups was significant (*p* < 0.05). Further pairwise comparison showed that the age of SCD (*plus)* was lower than the other two groups (aMCI and AD) at the corrected test level *p*′^1^ (*p* < 0.001).

For BMI, the difference among the four groups was also significant (*p* < 0.05). For a pairwise comparison, we only found the number of people with the BMI ≤23.9 Kg/m^2^ in NC group (39.1%) was smaller than that in the AD group, but no difference was shown between SCD (*plus*) group (46.7%) and AD group (78.4%) (*p* > 0.007).

A family history of dementia was presented in 26.7% of the SCD (*plus*) group. The proportion of currently smoking and drinking in SCD (*plus*) group was 10.0 and 16.7%, respectively. No significant differences among the four groups were found for family history of dementia, smoking and drinking (*p* > 0.05).

The proportion of having hypertension, cerebrovascular disease, cardiovascular disease, diabetes, and hyperlipidemia as multimorbidity in the SCD (*plus*) group was 40.0, 13.3, 6.7, 23.3, 36.7 and 30.0%, respectively, but again there were no differences between the SCD (*plus*) group and the other groups (*p* > 0.05) (Table 2).

**TABLE 2:** Comparisons between NC, SCD (*plus*), aMCI, and AD groups for clinical characteristics.

**Characteristics**	**Groups**				
	**NC (*n* = 23) N (%)**	**SCD (*plus*) (*n* = 30) N (%)**	**aMCI (*n* = 45) N (%)**	**AD (*n* = 37) N (%)**	***P*^#^**
Age, years					<0.01^*,**^
≤75	18(78.3)	29(96.7)	27(60.0)	22(59.5)	
>75	5(21.7)	1(3.3)	18(40.0)	15(40.5)	
Gender					
Males	12(52.2)	13(43.3)	19(42.2)	11(29.7)	0.36
Females	11(47.8)	17(56.7)	26(57.8)	26(70.3)	
Education, years					0.06
≤12	15(65.2)	16(53.3)	32(71.1)	31(83.8)	
>12	8(34.8)	14(46.7)	13(28.9)	6(16.2)	
BMI, Kg/m^2^					<0.01^∗∗∗^
≤23.9	9(39.1)	14(46.7)	31(68.9)	29(78.4)	
>23.9	14(60.9)	16(53.3)	14(31.1)	8(21.6)	
Mental workers	16(69.6)	23(76.7)	25(55.6)	15(40.5)	0.01^∗∗^
Lifestyle					
Current smoking	4(17.4)	3(10.0)	4(8.9)	2(5.4)	0.51
Current drinking	5(21.7)	5(16.7)	5(11.1)	3(8.1)	0.44
Family history of dementia	1(4.3)	8(26.7)	5(11.1)	8(21.6)	0.09
Comorbidities					
Hypertension	9(39.1)	12(40.0)	13(28.9)	14(37.8)	0.72
Cerebrovascular disease	3(13.0)	4(13.3)	8(17.8)	9(24.3)	0.61
Heart disease	2(8.7)	2(6.7)	4(8.9)	4(10.8)	0.95
Diabetes	6(26.1)	7(23.3)	7(15.6)	5(13.5)	0.53
Hyperlipidemia	3(13.0)	11(36.7)	16(35.6)	10(27.0)	0.21
Multimorbidity	7(30.4)	9(30.0)	13(28.9)	14(37.8)	0.83

In the last column of this table, *p*# illustrates the results for among-four-group comparison. Symbols in the brackets demonstrate whether there was difference for the pairwise comparison: ^*^represented a significant difference between the SCD (*plus*) group and the aMCI group; ^∗∗^represented a significant difference between the SCD (*plus*) group and the AD group; ^∗∗∗^represented a significant difference between the NC group and the AD group. SCD (*plus*), subjective cognitive decline (*plus*); NC, normal control; aMCI, amnesic mild cognitive impairment; AD, Alzheimer Disease Dementia; BMI, body mass index; Multimorbidity, accompanied with two or more diseases above.

### Comparison of Scores of Neuropsychological Assessments Between NC, SCD (*plus*), aMCI, and AD

The scores of AVLT-First Immediate Free Recall (median AVLT-IR1 scores = 6) and AVLT-Long Delay Free Recall (median AVLT-LR = 9) of SCD (*plus*) group were lower than those of NC group (median values of AVLT-IR1 and AVLT-LR 7 and 11, respectively) (*p* < 0.05). We found no difference of the total score of MoCA between the SCD (*plus*) group and the NC group, the median scores of which was 25.5 and 28.0, respectively (*p* = 0.08).

All the other scores of neuropsychological assessments, including AVLT-Second Immediate Free Recall (AVLT-IR2), AVLT-Third Immediate Free Recall (AVLT-IR3), AVLT-Recognition Recall (AVLT-RR), total scores of MMSE and MoCA, and single cognitive domain scores of MoCA test -the Trail Making Test (TMT), copy cube, clock drawing test (CDT), naming, digit span, alertness test, continuous subtraction 7, repeat sentence, Verbal Fluency Test (VFT), abstract test, MoCA-Delay Free Recall (MoCA-DR) and orientation test, were higher in SCD (*plus*) group compared to those in the aMCI and AD groups (*p* < 0.05). However, no significant differences were found between SCD (*plus*) and NC group (*p* > 0.05) (Table 3).

**TABLE 3:** Comparison between SCD (*plus*) and NC, aMCI and AD group for scores of neuropsychological assessments.

**Variables**	**Groups**						
	**SCD (*plus*) Percentile 50 (Percentile 25,75)**	**NC Percentile 50 (Percentile 25,75)**	**aMCI Percentile 50 (Percentile 25,75)**	**AD Percentile 50 (Percentile 25,75)**	***P* (SCD vs. NC)**	***P* (SCD Vs. aMCI)**	***P* (SCD Vs. AD)**
AVLT-IR1	6.0(4,7)	7.0(6,9)	5.0(4,5)	2.5(1,3)	0.04	<0.01	<0.01
AVLT-IR2	9.0(7,11)	10.0(8,12)	6.0(5,7)	3.5(2,5)	0.10	<0.01	<0.01
AVLT-IR3	11.0(9,13)	12.0(10,14)	7.0(6,9)	5.0(3,6)	0.06	<0.01	<0.01
AVLT-LR	9.0(7,11)	11.0(9,14)	2.0(0,5)	0.0(0,1.75)	0.03	<0.01	<0.01
AVLT-RR	12.5(10.75,14)	13.0(10,14)	7.0(5,10)	4.0(1,6.75)	0.64	<0.01	<0.01
MMSE	28.0(27,29.25)	29.0(27,30)	24.0(21,27)	17.0(12.25,21)	0.87	<0.01	<0.01
MoCA	25.5(25,27.25)	28.0(26,28)	18.0(15,22)	11.0(8,15.75)	0.08	<0.01	<0.01
TMT	1.0(0,1)	1.0(0,1)	0.0(0,1)	0.0(0,0)	0.82	<0.01	<0.01
Duplicate-C	1.0(1,1)	1.0(0,1)	1.0(0,1)	0.0(0,0.75)	0.39	<0.01	<0.01
CDT	3.0(3,3)	3.0(3,3)	2.0(1,3)	1.0(1,1.75)	0.54	<0.01	<0.01
Naming	3.0(3,3)	3.0(3,3)	3.0(2,3)	2.0(1.25,3)	0.07	0.03	<0.01
Digit span	2.0(2,2)	2.0(2,2)	2.0(2,2)	2.0(1,2)	0.07	0.01	<0.01
Alertness test	1.0(1,1)	1.0(1,2)	1.0(0,1)	0.0(0,1)	0.41	0.01	<0.01
Subtraction 7	3.0(3,3)	3.0(3,3)	3.0(2,3)	1.5(0,2)	0.10	<0.01	<0.01
Repeat-S	1.0(1,2)	2.0(1,2)	1.0(0,1)	0.0(0,1)	0.34	0.01	<0.01
VFT	1.0(1,1)	1.0(1,1)	1.0(1,1)	0.0(0,1)	1.00	0.02	<0.01
Abstract test	2.0(1,2)	2.0(1,2)	1.0(0,2)	0.0(0,1)	0.75	<0.01	<0.01
MoCA-DR	3.0(2,4)	3.0(3,4)	0.0(0,1)	0.0(0,0)	0.17	<0.01	<0.01
Orientation	6.0(6,6)	6.0(6,6)	5.0(4,6)	2.0(1,4)	0.78	<0.01	<0.01

*The *p* values represent the comparison between the SCD (*plus*) group and the other three groups (NC, aMCI, and AD). SCD (*plus*), Subjective Cognitive Decline (*plus*); NC, normal control; aMCI, amnesic Mild Cognitive Impairment; AD, Alzheimer Disease Dementia; AVLT-IR1, Auditory Verbal Learning Test- First Immediate Free Recall; AVLT-IR2, Auditory Verbal Learning Test-Second Immediate Free Recall; AVLT-IR3, Auditory Verbal Learning Test-Third Immediate Free Recall; AVLT-LR, Auditory Verbal Learning Test-Long Delay Free Recall; AVLT-RR, Auditory Verbal Learning Test-Recognition Recall; MMSE, Mini-Mental State Examination; MoCA, Montreal Cognitive Assessment; CDT, clock-drawing test; TMT, Trail Making Test; Duplicate-C, duplicate cube; Repeat-S, repeat sentence; VFT, verbal fluency test; MoCA -DR, Montreal Cognitive Assessment-Delay Free Recall.*

### Affecting Factors for NC, SCD (*plus*), aMCI and AD Groups

Our results of the multiple logistic regression analysis showed that aging, years of education, job category and BMI were affecting factors of SCD (*plus*). Aging was an important risk factor for SCD (*plus*) progressing to aMCI (OR = 0.05, 95% CI = 0.01–0.41) and AD (OR = 0.03, 95% CI = 0.01–0.39), which also showed a certain risk effect on the progression of NC to SCD (*plus*) subjects (OR = 0.10, 95% CI = 0.01–0.93) (*p* < 0.05). Mental work had a protective effect on SCD (*plus*) progressing to AD patients (*p* < 0.05), whereas lower education (OR = 4.43, 95% CI = 1.03–19.18) and lower BMI (OR = 3.73, 95% CI = 1.08–12.98) were significantly associated with greater odds of SCD (*plus*) progressing to AD patients (*p* < 0.05) (see Table 4).

**TABLE 4:** Affecting Factors for NC, SCD (*plus*), aMCI and AD groups.

**Characteristics**	**NC**	**aMCI**	**AD**
	**OR (95% CI)**	**OR (95% CI)**	**OR (95% CI)**
Age, years			
≤75	0.10(0.01–0.93)	0.05(0.01–0.41)	0.03(0.01–0.39)
>75	Ref.	Ref.	Ref.
Education, years			
≤12			4.43(1.03–19.18)
>12	Ref.	Ref.	Ref.
Job category			
Mental work			0.25(0.06–0.98)
Physical work	Ref.	Ref.	Ref.
BMI			
≤23.9			3.73(1.08–12.98)

*NC, normal control; aMCI, amnesic Mild Cognitive Impairment; AD, Alzheimer Disease Dementia; OR, odds ratios; CI, confidential interval. Ref., this group was used the reference group.*

### ROC of NC and SCD (*plus*)

We used the ROC curves to evaluate the goodness of the affecting features and neuropsychological scores, respectively, on discriminating the SCD (*plus*) group from the NC group. As a variable that differed significantly between all the groups, we first used age as the factor and found its AUC was 0.592 (95% CI, 0.434–0.750), which was low. To further explore the optimal discriminating model, we continued to add more factors and we found that by using age, gender, years of education, job category, BMI, current smoking, and current drinking, the AUC reached 0.673 (95% CI, 0.524–0.823).

**FIGURE 1:**
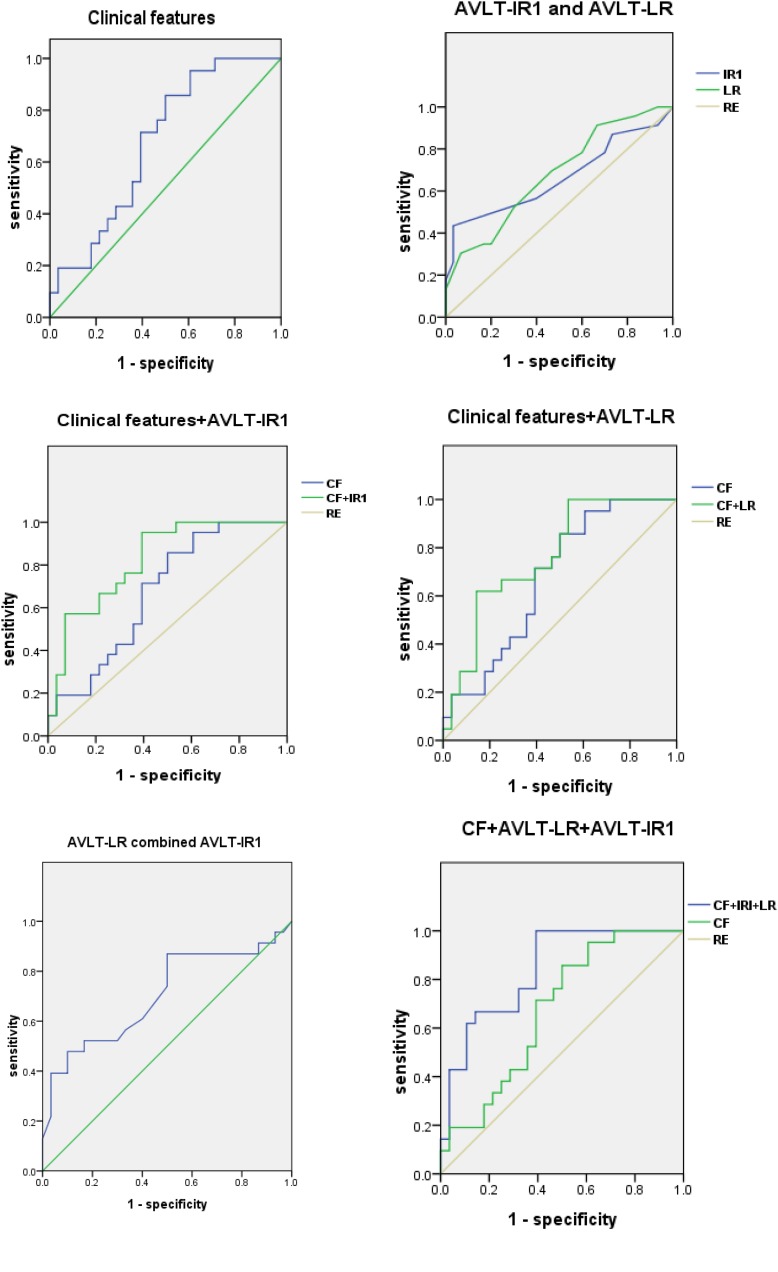
ROCs for NC and SCD (*plu*s) group. ROC curves for NC and SCD (*plus*) group using clinical features, AVLT-IR and AVLT-LR. AVLT-IR1, Auditory Verbal Learning Test-First Immediate Free Recall; AVLT-LR, Auditory Verbal Learning Test-Long Delay Free Recall; ROC, receiver operating characteristic; CF, clinical features; RE, reference.

Based on the results of the neuropsychological assessments, we also performed ROC analysis by using scores of AVLT-LR and AVLT-IR1 as variables, and the AUCs were 0.679 (95% CI, 0.535–0.823) and 0.662 (95% CI, 0.506–0.819), respectively. Finally, we added the clinical features including the demographics and life styles as variables in the logistic regression in addition to AVLT-LR or AVLT-IR1, respectively, and we found that the AUC increased to 0.823 (95% CI, 0.708–0.938) and 0.764 (95% CI, 0.631–0.897). When combining AVLT-IR1, AVLT-LR and clinical variables in the regression model, the AUC increased from 0.673 to 0.838 (95% CI, 0.729–0.948) (Figure 1).

## Discussion

To the best of our knowledge, the risk of progressing to AD is higher for SCD (*plus*) than for SCD, but there have been few studies reporting its risk factors and neuropsychological assessment characteristics. This is the first study that reveals the presence of early episodic memory impairment in SCD (*plus*) population in memory clinic by fulfilling all the six items of the diagnostic framework. This study also characterized the clinical features and neuropsychological assessments of SCD (*plus*) in relative to normal controls, amnestic mild cognitive impairment (aMCI) and Alzheimer disease (AD).

We identified lower age, longer education period, and more mental work as the demographic characteristics of SCD (*plus*) group, compared with aMCI and AD group, which are in agreement with the previous studies (Mohs et al., 2001; Sando et al., 2008; Hebert et al., 2010; Iliffe and Pealing, 2010; Jefferson et al., 2011; Meng and D’Arcy, 2012; Alzheimer’s Association, 2014; Beydoun et al., 2014; Garcia-Ptacek et al., 2014; Liang et al., 2018). We also showed that higher BMI was a protective factor for SCD (*plus*) progressing to AD patients, which was consistent with some of the previous studies (Barnes et al., 2009; Fitzpatrick et al., 2009; Xu et al., 2011; Gustafson and Luchsinger, 2013; Qizilbash et al., 2015). Latent AD may be accompanied by the metabolic changes that are not yet fully understood. Yet this phenomenon may be associated with the changes of body composition with aging, such as the morphological changes of fat cells and the adipose tissue compartment, diminished muscle mass, sarcopenia, and somatic frailty. For women who are affected by the reproductive aging and changes in the sex hormone milieu, it may be related to the alteration of adipose tissue. On the contrary, other researchers argued that obesity increased the risk of dementia (Beydoun et al., 2008; Profenno et al., 2010; Sellbom and Gunstad, 2012; Loef and Walach, 2013), which is also greatly correlated with other morbidities, such as hypertension and diabetes (Luchsinger and Gustafson, 2009). These inconsistencies may be attributed to the error in the measurement of adiposity. Besides, other factors (Devore et al., 2010; Littlejohns et al., 2014; Emmerzaal et al., 2015; Qizilbash et al., 2015), such as cholesterol levels, age-related regulatory changes in carbohydrate, lipid or protein metabolism, increased intake of vitamin E, anti-oxidant and vitamin D may all affect the relationship between BMI and dementia. It is also possible that the higher BMI was the result of having olfaction firstly affected in the progression of AD (Gustafson and Luchsinger, 2013).

Smoking was regarded as a risk factor of dementia (Lee et al., 2010; Beydoun et al., 2014; Zhong et al., 2015), but we found no statistical differences between all the groups, which may be due to the low percentage of smokers in our groups. Also, there was no significant correlation between alcohol consumption and cognitive impairment, which was in line with previous findings (Baumgart et al., 2015).

Earlier studies have found that hypertension in later life was a protective factor for cognitive decline (Lee et al., 2010; Beydoun et al., 2014), whereas diabetes, hyperlipidemia and cerebrovascular disease increased the risk of dementia (Honig et al., 2003; Profenno et al., 2010; Gudala et al., 2013; Roberts et al., 2014; Cooper et al., 2015). In our study, we did not find significant difference of comorbidities including hypertension, cerebrovascular disease, cardiovascular disease, diabetes and hyperlipidemia between groups, and this inconsistency might be due to the following reasons: (1) Those chronic diseases were not stratified according to the sex, disease duration and severity. A few studies showed that middle-aged individuals with hypertension and diabetes for longer than 6 years had a positive correlation with cognitive decline (Helzner et al., 2009), Sex difference in the presence of comorbidities which involve the vascular contributions to the cognitive impairment and dementia should be considered (Gannon et al., 2018); and (2) our patients did not co-present as many diseases as they were shown in one previous study, such as arthritis, prostate disease, lung disease and so on (Aarts et al., 2011).

In our study, after controlling the clinical characteristic variables, the SCD (*plus*) group showed lower scores of AVLT1 and AVLT-LR than those of the NC group (*p* < 0.05). This suggests that memory impairment has already presented in SCD (*plus*) population at the AD preclinical stage. We also showed that the combination of AVLT-IR1 and AVLT-LR improved the diagnostic accuracy of SCD (*plus*) compared to the condition when they were used separately, which indicates that AVLT may allow for distinguishing SCD (*plus*) form NC individuals. It also suggests that in order to better identify SCD (*plus*), episodic memory should be included as part of the neuropsychological assessment. Delayed recall in AVLT is considered to be the most sensitive measure of early AD (Zhao et al., 2012). However, not all studies have included this test (Yang et al., 2015). Our SCD (*plus*) individuals showed slightly worse performance on the challenging cognitive tasks than individuals without cognitive complaints (Koppara et al., 2015; Smart and Krawitz, 2015). Also, compared to no complaints, reduced episodic memory learning effect and poorer performance on psychomotor speed and language were present in SCD participants (Kielb et al., 2017). However, a different cutoff value from what is currently used for discriminating MCI from NC may need to be provided in the future to differ SCD (*plus*) from NC with better sensitivity and reliability. For the global cognition revealed by MoCA and MMSE, although the differences between the SCD (*plus*) and NC groups were not significant, MoCA showed a higher sensitivity of assessment compared with MMSE. Thus, MoCA may be helpful to be included in the SCD (*plus*) screening scales, but further verification is needed by follow-up studies.

In addition, our study found that except for memory impairment, the other cognitive domains also had begun to decline from the aMCI stage (*p* < 0.05). It indicates that once a patient enters the stage of MCI, other cognitive domains may also have been damaged. Deng (2014) found that 54.2% elderly in the a different cohort had cognitive impairment, and the abstract scores were lower in both the normal control and cognitive impairment groups. The memory impairment group scored lower in the domains of execution, visual space, language and delayed recall. MCI is often characterized by slight but noticeable deficits in attention, learning and memory, executive function, processing speed, and semantic language (Storandt et al., 2006; Saunders and Summers, 2011; Summers and Saunders, 2012), and the early cognitive impairment of these domain are also strong predictors of the progression from MCI to AD (Brandt et al., 2009; Klekociuk et al., 2014). In order to be sensitive to the impairment of single cognitive domain in SCD (*plus*), questionnaires designed for screening patients with cognitive impairments need to report not only the global cognition scales (such as MMSE, MoCA), but also the scores of single cognitive domains, such as language and execution etc.

The limitations of this study are: (1) our sample size is relatively small, which might be the cause of some of the negative results between groups. Further investigations with larger sample sizes are needed; (2) Our study is a cross-sectional survey and follow-up studies should be performed to further confirm the final conclusions; (3) We adopted the standardized criteria of pre-MCI SCD proposed in 2014 by Jessen (Jessen et al., 2014) given depression and anxiety maybe the early presentations of the SCD (*plus)*. We admit that using this criterion, some of the subjects would have mild to moderate anxiety and depression. However, the present study did not address this issue, which would be of interest to study further. (4) The diagnosis of SCD (*plus*) was not validated by the other tests. For instance, it lacks the completeness of Aβ-PET, APOEε4, cerebrospinal fluid tau or Aβ examinations, given that only ∼60% of the included population had genetic tested and Aβ-PET undertaken; and (5) finally, other related biomarkers and imaging approaches need to be investigated to gain more understanding of SCD (*plus*).

In summary, we characterized the SCD (*plus*) and unraveled that aging, shorter education period, physical labor work and lower BMI are risk factors for SCD (*plus*) progressing to aMCI or AD. This study may provide a reference to the inclusion criteria for the future early interventional studies and may pave the way for exploring more sensitive neuropsychological assessments for the cognitive decline in SCD (*plus*) individuals.

## Data Availability

All datasets generated for this study are included in the manuscript and/or the supplementary files.

## Ethics Statement

This study was approved by the Medical Ethics Committee of Xuanwu Hospital of Capital Medical University, Beijing, China. Written informed consent was obtained from either participants or their legally agreed surrogates.

## Author Contributions

All authors listed have made a substantial, direct and intellectual contribution to the work, and approved it for publication.

## Conflict of Interest Statement

The authors declare that the research was conducted in the absence of any commercial or financial relationships that could be construed as a potential conflict of interest.

## Footnotes

1 *p*′ = 0.05/[*k*(*k*−1)/2 + 1] (*k* = 4), α′ = 0.007.

## Appendix

### Pre-MCI SCD

It is defined as (1) the objective cognitive performance of the individuals to be within the normal range; (2) decline of the self-perceived cognitive abilities; (3) these subtle cognitive declines should not reach the diagnosis of mild cognitive impairment (MCI) or dementia; and (4) this should not be caused by any psychiatric disorders or neurological and medical conditions.

### SCD (*plu*s)

In addition to meeting the diagnosis of pre-MCI SCD, SCD (*plus*) also need to meet the following criteria: onset age ≥60 years; complaints about SCD within the past 5 years; the complainers feel their performance are not as good as their peers and concerning associated with SCD; a confirmed cognitive decline by the informants; complains were only limited memory problems rather than other cognitive domains; and presence of the APOE ε4 genotype and biomarker evidence for a potential progression to AD.
